# Human Genomics and the Biocultural Origin of Music

**DOI:** 10.3390/ijms22105397

**Published:** 2021-05-20

**Authors:** Livia Beccacece, Paolo Abondio, Elisabetta Cilli, Donatella Restani, Donata Luiselli

**Affiliations:** 1 Laboratory of Molecular Anthropology, Department of Biological, Geological and Environmental Sciences, University of Bologna, 40126 Bologna, Italy; livia.beccacece@studio.unibo.it; 2 Department of Cultural Heritage, University of Bologna—Ravenna Campus, 48121 Ravenna, Italy; elisabetta.cilli@unibo.it (E.C.); donatella.restani@unibo.it (D.R.)

**Keywords:** music, musicality, genetics, evolution, expression, adaptation, culture

## Abstract

Music is an exclusive feature of humankind. It can be considered as a form of universal communication, only partly comparable to the vocalizations of songbirds. Many trends of research in this field try to address music origins, as well as the genetic bases of musicality. On one hand, several hypotheses have been made on the evolution of music and its role, but there is still debate, and comparative studies suggest a gradual evolution of some abilities underlying musicality in primates. On the other hand, genome-wide studies highlight several genes associated with musical aptitude, confirming a genetic basis for different musical skills which humans show. Moreover, some genes associated with musicality are involved also in singing and song learning in songbirds, suggesting a likely evolutionary convergence between humans and songbirds. This comprehensive review aims at presenting the concept of music as a sociocultural manifestation within the current debate about its biocultural origin and evolutionary function, in the context of the most recent discoveries related to the cross-species genetics of musical production and perception.

## 1. Introduction

Since ancient times music is paramount in many social and cultural activities, such as rituals, education and performances, included choreutics [1]. Together with language, it is present in all populations and cultures, around the world and over time: no human group has been found, that does not bear at least a rudimentary form of music and verbal communication [1,2]. Thus, these two forms of communication could be also useful to highlight the patterns of human migration and cultural admixture [3].

Humans are able to perceive and define intrinsic characteristics in music, such as structure, rhythm, intensity, pitch, and timbre [4]. Moreover, they have the capacity to entrain to an isochronous pulse (co-coordination of movements to a rhythmic pulse), an ability required for music production as well as vocal learning, or the skill of learning new melodies [3,5,6,7]. Excluding humans, a phenomenon similar to music is referred to as “vocalizations”, such as those of songbirds, although these are thought to be functionally limited to mate attraction and territoriality [8].

However, what is music? It can simply be described as a periodic variation of pitches, which, when combined, originate melodies. Nevertheless, a definition of music is nontrivial and may be different in distinct cultures, although it always underlies a form of universal communication that awakens an emotional response [7,9]. In any case, it is possible to introduce a first distinction between music and musicality. Musicality can be defined as a set of traits or abilities that allows us to perceive and produce any form of music, that has a spontaneous development and is constrained by cognitive and biological systems. Music, indeed, is a sociocultural concept based on that of musicality, in other words it is a product of musicality, that varies among cultures and over time [10].

The reconstruction of the spread and evolution of music, however, is not easy and is challenged by the paucity and fragmentary conditions of archeological records. To date, the oldest artifacts undoubtedly recognized as musical instruments are described as pipes and flutes, recovered from the sites of Geißenklösterle, Hohle Fels and Vogelherd in Germany [11], which date back to 43,150–39,370 years before present (BP) [12]. There is also an ongoing debate about an osseous artifact of similar age, recovered in 1995 from the Divje Babe I cave site in Slovenia and belonging to the Mousterian techno-complex of Western Eurasia, arguably providing evidence for the active manipulation of bone to obtain wind musical instruments in Neanderthals [13,14,15]. It is also noteworthy that the sound produced by a seashell horn has been analyzed and recorded for the first time just recently. This seashell, forgotten for many years and not considered until now a music instrument, shows signs of human modifications, such that it can be classified as wind instrument. It was found in a cave in Marsoulas (France), and it is attributed to the early Pyrenean Magdalenian culture of the Upper Paleolithic (around 18000 years cal BP). These results make it the oldest seashell horn of the Upper Paleolithic [16].

Despite the lack of ancient artifacts, a cross-cultural approach highlighted universal features of music, for example related to pitch and rhythm; furthermore, some of these features have parallels in non-human animals, such as songbirds [2]. The presence of similarities across cultures supports musicality as a significant characteristic of humankind and suggests underlying cognitive and biological mechanisms that may constrain and shape music across cultures [9]. Therefore, these universal music features can be considered as the ‘musical possibilities’ of the human being and are based on an anthropological and phylogenetic substratum, which guarantees the existence of cultural universals [17].

## 2. Music–Language Universality and the Biocultural Origins of Music

Another debate arises around the evolutionary relationship between music and language, since both possess emotional content, involve change of pitch, timbre, loudness, rhythm processing [18,19,20], are rule-based and can be transmitted in written form [2,3]. It has been proposed that music and language have originated from a common precursor called *musilanguage* (or protolanguage) and then have differentiated by developing distinct properties [21]. Since vocal learning is a feature shared by both language and music, it is possible to argue that this characteristic emerged at the same time of protolanguage; so, its appearance would also mark the passage from innate to learned vocalization [22]. This common origin could be supported by positron emission tomography (PET) experiments, which show a partial overlap between brain areas activated by music and by language, such as Broca’s area, the prefrontal cortex and the amygdala [23,24].

The universality of music and its parallels with language raise questions around its origin and function: how and when the ability to appreciate and produce music evolved in our species? Its general features suggest that anyone has the potential to engage in music-related activities [25]. Indeed, music may be perceived as a biological competence, given that humans are responsive to it from a very early age [21]. A fundamental debate among scientists relates to the possibility that music, then, originated as an evolutionary adaptation, rather than a cultural product [26]: if music can be treated as a biological trait under selective pressure, it may have played a role in the survival of the human species [9,27]. It has been proposed, in fact, that music is a biological adaptation with at least three different roles: the increase of reproductive success, strengthening mother–infant connection and social cohesion.

The first hypothesis, dating back to the earliest observations by Charles Darwin that animal vocalizations were of benefit for the reproductive success of the individual, rather than its survival [28], has been recently corroborated by studies on the audio–visual integration effect of music-elicited emotions in humans: a cross-modal transfer of musical arousal seems to be responsible for increased levels of attraction towards opposite-sex faces in women, and a congruent audio–visual presentation (pleasant music and a face with sexually attractive characteristics) induces a stronger physiological response [29,30,31].

The second hypothesis considers that cognitive skills and emotional sensibility to music may have evolved gradually from affective interactions between ancestral mothers and their infants since approximately two million years ago, involving vocalizations with proto-musical features; mother–infant vocalization was, then, an adaptive character that allowed to increase the survival of infants and, therefore, the reproductive success of the mothers [32,33]. At a later time, the elements originating from mother–infant vocalizations became music [34]. Reconnecting to the musilanguage theory [21], it may be speculated that the prosodic maternal–infant communication is also a proxy for the communication system used by our ancestors, prior to the separation of music and language [18,19].

The third adaptive hypothesis argues that music, and consequently language, evolved to enhance cohesion in increasingly larger social groups [20]. Humans have a cross-cultural and universal propensity to gather in groups to sing and dance rhythmically together, often in a ritual setting; this behavior does not seem to generate an immediate benefit for the survival of the human species and the biological background of this tendency may be searched in the social displays that are typical of the apes, such as “loud calls” and physical manifestations [5,6]. Social interactions and cohesion are facilitated by music, as they are associated with common physiological arousal states in individuals who assist to musical performances, therefore promoting the expression of emotions [3,34]. A significant support to this theory could be provided by the overlap between brain regions activated when listening to music and when performing cooperative tasks, and also by the neurochemical and physiological evidence that both events stimulate dopamine release as a rewarding response [19,35]. The nucleus accumbens, which has a role in this response during listening to music [35], is also one of the brain regions activated when individuals are performing reciprocally collaborative tasks that are rewarding and empathic [36,37], corroborating the hypothesis of music as “social glue”. There is, indeed, direct evidence not only for the causal role of dopamine in pleasure response when listening to music as compared to highly rewarding hedonic experiences, but also for the stimulation of oxytocin and the consequent inducement of prosocial behavior [38,39,40,41]. Neuroimaging, neuropsychological and brain stimulation studies in musical anhedonia (the inability to find music pleasurable) also seem to suggest that human appreciation of music emerges from the predictive process of auditory perception, which connects to the structures involved in the brain’s dopaminergic reward system, to the extent that it may function as a stimulus for verbal episodic memory and learning [42,43,44].

Laurel J. Trainor [45] suggests that the adaptive benefits of musical abilities are not obvious and that the rapid changing of music points towards strong cultural dependencies and, indeed, an undeniable cultural origin for music [45]. However, it is also undeniable that the emotional and social power of music may have determined benefits for the survival of the group and eventually led to adaptations. The article concludes that the origins of music are highly complex and probably involved, among others, exaptation from language, cultural creation and evolutionary adaptation at different times and in different social groups or populations [45,46,47,48,49,50,51].

**Figure 1:**
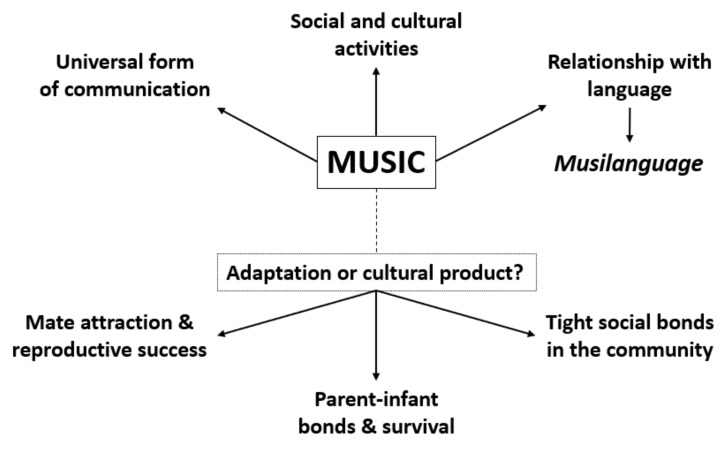
Schematic representation of the hypothesis of the evolution of music.

Figure 1 shows the hypothesis on the evolution of music discussed above.

## 3. Comparative and Cross-Species Behavioral Studies

Despite the absence of fossils from a remote past not allowing to provide a physical evidence for the history of musicality, comparative research can be a very useful approach to learn about the origins of music and can provide evidence of the adaptive roles of this trait in other species. This can be done by focusing on the capacities which underlie musicality, especially by studying animals that share specific musical characteristics with humans, such as complex vocalizations, entrainment and beat perception [1,9]. These comparative studies, mostly consisting of behavioral experiments, can provide insights about the neural activity involved in musicality [51,52]. Among the species studied with a comparative approach, *Macaca mulatta* (rhesus monkey) and *Taeniopygia guttata* (zebra finch) are much employed animal models about auditory system, speech and the brain structure [1]. Moreover, in non-human animals the ability to entrain movements to an auditory rhythmic beat has been found in parrots (*Cacatua galerita*, [53]), sea lions (*Zalophus californianus*, [54]), chimpanzees (*Pan troglodytes* sp. [55,56,57]) and bonobos (*Pan paniscus*, [58]). Despite this, humans are the only which possess a motor synchronization based on temporal anticipation with a mental model of time [59,60,61].

The finding that the skill to entrain to a rhythmic pulse is not exclusive of humans could give support of the idea of Darwin that the perception of rhythm may be a shared trait in animals [62,63], although other theories have been introduced in the last two decades. Briefly, the vocal learning and rhythmic synchronization hypothesis [64] states that beat perception and synchronization are a by-product of mechanisms underlying vocal learning, which is prerequisite for the ability to move in time with complex musical rhythms; nevertheless, the evidence of entrainment in a sea lion [54], which is not a vocal learner, challenges this hypothesis. Moreover, several recent studies on rhesus monkeys highlight the difficulty to synchronize their movements to complex auditory and visual stimuli, and the preference for a visual cue to drive their movements [65,66,67]. The gradual audiomotor evolution (GAE) hypothesis [68] suggests, instead, that rhythmic entrainment has developed through a gradual series of changes, both anatomical and functional, mainly in the brain of primates, without vocal learning being essential in this context. Studies on chimpanzees and bonobos show that they naturally exhibit a rhythmic behavior through body movement and drumming on objects (although not accurately aligned to an auditory rhythm and appearing regardless of regularity in beat); this suggests that the last common ancestor to humans and chimpanzees could have possessed fundamental characteristics for music, while sexual dimorphism in sensitivity to auditory stimuli in chimpanzees may indicate a characteristic acquired after the divergence from the common ancestor with humans, which do not show sex differences in music ability [55,56,57,69]. As a confirmation of this hypothesis, the motor cortico-basal ganglia-thalamo-cortical (mCBGT) circuit, which is involved in beat perception [70], shows to be more developed in humans than non-human primates [71,72].

## 4. Twin Studies, Heritability and Hints to the Neuropsychology of Music

Twin studies have been extensively used to dissect the heritability of behavioral and cultural traits, as they allow to estimate environmental and genetic influences on traits with the use of identical (monozygotic) or non-identical (dizygotic) twins, and extend these results to the general population. In the context of musical aptitude and music achievement, studies on a large cohort of Swedish twins (*n* = 10.975) have shown moderate genetic heritability for both traits, with estimated values up to 57% [73. The same study also tested for associations between musical aptitude and achievement and phenotypic traits descriptive of mating success, finding either nonsignificant or reverse associations that do not support the theory of music abilities enhancing mate attraction and reproduction [73].

The relationship between music, verbal ability and the capacity to decode symbolic information has been also analyzed through twin studies. Results show that musical aptitude and music training positively correlate with verbal abilities, an association mostly explained by shared genetic factors (50%) and non-shared environmental influences (35%) [74]. In a twin and adoption study performed on adolescents, it is shown that music engagement, dance and singing are all heritable (h = 78%, 66% and 43%, respectively) and that correlation between instrument engagement and verbal ability at 12 years of age is predictive of the same relationship at 16 years of age, with possible direct benefits of musical practice in later language abilities [75]. Interestingly, music is not only associated with increased speech perception, but recent twin studies seem to provide evidence for heritability and a positive relationship between sight-reading music ability and reading passage comprehension, suggesting an influence of decoding mechanisms that are independent of working verbal memory [76].

Other than twin studies, there has been growing interest in the neuropsychological analysis of subjects with congenital amusia (the inability to process and recognize music) and language impairments. These studies show that there is a strong correlation between language and music related to the development of skills in these two domains and their cognitive processing, independent of formal musical training [77,78,79]. Other researchers highlight how music perception influences language acquisition [80], is linked to spatial processing, visual memory and speech perception [81,82,83] and how the structural redundancy of speech and musical communication shapes the individual’s perceptual abilities [84,85]. Moreover, neuroimaging experiments document how music and language are processed in the same areas of the brain and in similar ways, on the basis of their syntactic integration mechanisms [86,87]. These observations suggest an intimate and intricate relationship between music and language abilities, as well as a neuroarchitectural overlap in their symbolic processing.

## 5. Genetic and Genomic Candidates for Musicality in Humans

Overall, humans show a musical aptitude, consisting of several skills such as discrimination of pitch, tone duration and musical memory [4]. The ascertained variability in this ability, starting from people unable to appreciate and engage with musical activities (congenital amusia) to virtuoso musicians [88,89], suggest a genetic basis of musicality. It is also well known that musicianship clusters in families, raising the question of the role of environmental factors besides to specific genetic variants [90,91].

In this context, genomic approaches allow to identify the genes associated with a specific trait (music, in this case) in an impartial way and without a priori hypothesis [92]. In the last decades, genome-wide studies have been carried out to find the genetic basis of musicality and the genetic variants associated with it, even if the bulk of these studies has been performed on Finnish individuals [93,94,95,96,97,98,99,100,101]. The results show that musical aptitude and creativity display a percentage of heritability [93,94], and that there are several genetic loci linked to musical aptitude, mainly including genes involved in neurocognitive functions, auditory pathways and the development of the inner ear.

In candidate gene studies, the first genes to have been related to musical aptitude are *AVPR1A* and *SLC6A4*, encoding, respectively, the arginine vasopressin receptor 1A and the serotonin transporter (5-HTT) [101,102,103,104,105,106,107,108,109,110]. Haplotypes of microsatellites (RS1, RS3) in the promotor region of *AVPR1A* were associated with dance performance [101], active listening to music [95], musical aptitude and creativity in music [94,103]. *AVPR1A* is involved in the control of higher cognitive functions such as memory and learning [103] and seems to play a role in several social behaviors, such as altruism [104] and autism [105]. *SLC6A4*, instead, was related to dance performance, together with *AVPR1A* [102], short-term music memory [106] and choral singing [96]. It is expressed in brain areas implicated in emotions and in reward-seeking behavior [107,108] and is involved in neuropsychiatric disorders and depression [109,110]. Therefore, these two genes could be good candidates since one of the adaptive roles of music is as “social glue” [20].

Overall, several genetic loci and specific variants have been identified, but the most significant evidence of linkage to musical aptitude has been found on chromosome 4p15-q24, both in Finnish and Mongolian populations [93,97,99]. This extended region contains several genes that could be involved in musical abilities [91,93,97,99,111]. The overlap of the results in populations of different ancestries (Finnish and Mongolian) increases the confidence of the association of this region with musical ability. Among the genes showing an association with musicality there are *UNC5C* [94], *UGT8* [98], *PCDH7* [99] and *SNCA* [111]. *UNC5C* (4q22) is a strong candidate thanks to the overlap of associations in Finnish [93] and Mongolian populations [98]. It encodes a netrin receptor (Unc-5 netrin receptor C) which, interacting with netrins, is implicated in axon extension and cell migration during the development of nervous system. It is interesting to note that netrins interact also with ROBO family receptors [112], one of which (ROBO1) is implicated in dyslexia [113], reconnecting thus to a probable evolutionary relationship between music and language.

In the Mongolian population SNPs of *UGT8* gene, located on 4q23, showed the strongest association with musicality [108]. It encodes UDP glycosyltransferase 8, highly expressed in the brain and involved in in the biosynthesis of complex lipids in myelinating oligodendrocytes and in the clearance of long-chain ceramides [114].

Another probable candidate gene localized on chromosome 4 is *PCDH7* [99], encoding the protocadherin 7. Like other protocadherins, it is highly expressed in the brain and seems to be involved in brain development and neuronal survival, but the cellular mechanisms in which is implicated are not yet understood [115]. Instead, in model animals, it is shown to be implicated in the development of the cochlea in chicken [116] and expressed in the amygdala in mice [117]. In particular, the amygdala is the brain area involved in the emotional processing of music [118], making this gene a good candidate for musical aptitude.

Evidence of linkage was found also on other chromosomes, such as 18q [93]: this region overlaps with the *DYX6* locus, a gene associated with dyslexia [119], hinting at the common origin of language and music.

Several copy number variations (CNVs) also showed association with musical aptitude and a creative phenotype. For example, a duplication on 2p22.1, a genomic region that contains the *GALM* gene, has been related to creativity through the implication of this gene in the uptake of serotonin in the thalamus [96]. A deletion on 5q31.3, in which is located the gene *PCDHA 1–9*, showed association with low scores in musical aptitude tests [96]. The protocadherin-α gene cluster consists of 14 genes arranged in tandem [120], which encode neuronal adhesion proteins involved in synaptogenesis and maturation of serotoninergic projections in most brain areas [121], learning, and memory [122]. Interestingly, a 1.3 Mb duplication associated with low scores of musical aptitude tests has been also identified on 8q24.22 [96], overlapping with the region previously linked with absolute pitch in families with European ancestry [123]. The results of this genome-wide copy number analysis [96] overall show that several CNVs regions associated with significant scores of musical aptitude test contain genes involved in neuropsychiatric disorders.

Recently, the first genome-wide association study was published associating 67 loci at genome-wide significance (*p*-value < 10^−8^) with self-reported beat synchronization in a large cohort of more than 600,000 individuals, suggesting that this phenotype is a highly polygenic trait with shared genetic architecture to breathing, motor function, processing speed and chronotype [124].

Debate remains on whether music was subjected to positive selection. A study performed to identify genomic regions under positive selection associated with musical aptitude found several candidate regions, but no specific genetic targets. However, among the genes contained in these regions, several are involved in brain functions and hearing, but also in human language development and cognitive disorders, which makes them plausible candidates [120].

Summarizing, the results of genome-wide linkage analysis suggest several candidate genes for musical ability (Table 1), indicating that there is a basic, common musical competence in humans and that music is a complex, polygenic trait. The genes associated with musicality are involved in cognitive functions, neurodevelopment, neuronal plasticity, inner ear development, dopamine release and transport, and singing in songbirds; many have also been related to psychiatric disorders and neurodegenerative diseases. Therefore, musical aptitude seems to be the result of several genomic variations, for the most part still unknown, and complex gene–environment interactions. Two review papers identified many top candidate genes associated with musicality, integrating the results of a wide range of previous genetic and comparative studies on humans and other animals [91,111]. The involvement of several of these genes in singing and song learning in songbirds and in human musicality demonstrates an evolutionary convergence in recognition and production of sounds between humans and songbirds, and thus a common molecular basis of musical features [111]. In general, the top ranked genes in these studies show involvement in cognition, memory, learning, neuronal excitation and apoptosis, long-term potentiation and neuronal plasticity. However, the current knowledge on genetic pleiotropy and the small contributions of hundreds of genes to single traits (polygenic and omnigenic models) urges for the use of genome-wide methodologies, rather than candidate gene studies, to better disentangle the genetic contributions to music ability in humans.

## 6. The Effect of Music on Gene Expression and Brain Activity

Each individual, when listening to music, experiences different emotions, such as calm or enthusiasm, and generally feels a sensation of wellbeing. In fact, using positron emission tomography (PET) it has been determined that music-evoked pleasure is associated with dopamine secretion in the mesolimbic reward centers, mainly in the right caudate and the right nucleus accumbens located in the ventral striatum. This dopaminergic activity results in emotional arousal, leading to rewarding feelings [35,140], and perhaps it also involves the opioid circuit with the release of endorphins, as occurs in primary rewards [38].

On the other hand, many studies showed that musical training has several effects on musicians’ brains, both in structure and function [141,142]. For example, musicians have a greater volume in the auditory cortex [143], enhanced synaptic plasticity [144], enhanced cognitive performances, visual attention abilities, verbal and long-term memory, and reasoning [126,145,146].

At molecular level, it has been shown that both listening to and playing music determine epigenetic alterations, by mediating a differential expression of several genes and microRNAs (miRNAs) [126,127,146,147]. Of note, there is overlap between the results of microRNAs and those of genes differentially expressed; in particular, some of these genes are target of the miRNAs with differential expression, both regarding the conditions of music performance and listening to music. In this body of research, samples of peripheral whole blood were used, as its transcriptome is shared for more than 80% with other tissues, including the brain which is inaccessible [148,149,150,151]. It has been verified that, for professional musicians, a concert of two hours induces a differential expression of 73 genes, involving both upregulation (51 genes) and downregulation (22 genes) [127]. In an analogous way, listening to music determines the differential expression of several genes and microRNAs, but depending on the degree of musical education and musical aptitude [147,148]. It is interesting to note that some of the genes are regulated both after music performance and listening to music and there is also evidence that some genes are upregulated after music performance but downregulated after listening to music [147,148]. Both after listening to and playing music, the upregulated genes are involved in comparable functions, such as the neuronal homeostasis of dopamine, cognitive functions, neuronal plasticity, learning and memory [147,148]. On the other hand, the downregulated genes include the ones targeted by transcription regulators for proinflammatory cytokines [147] and in neuronal apoptosis [148]. Regarding the changes in microRNAs transcriptome, playing and listening to music induce the up- and downregulation of different microRNAs, and some of these are involved in the same pathways/functions. It has been found that some of the upregulated microRNAs after both experiences target genes involved in negative regulation of neuronal apoptosis and inhibition of the cell cycle, suggesting a neuroprotective role of music, and in song perception and production in songbirds (for example, *FOXP2*) [148,149].

Overall, the results of these studies show a correspondence between the upregulated and downregulated biological functions after listening to and music performance. Interestingly, several differentially regulated genes and microRNAs are involved in song learning and singing in songbirds. Indeed, some of the genes and microRNAs differentially expressed [127,147,148] have been identified as essential for auditory perception and speech production in both humans and songbirds [128]. For example, among the upregulated genes after music performance, *SNCA*, *FOS*, and *DUSP1* have been identified as biomarkers of song perception and production in songbirds [128,129,152,153,154]. Therefore, these results might indicate an evolutionary convergence in sound perception and vocal communication between songbirds and humans.

The alpha-synuclein gene (*SNCA*) is located in a candidate region for musical aptitude on chromosome 4q22.1 [92,97,98] and is regulated by GATA2 protein [133]. *GATA2* (3q21.3) is in turn one of the best associated genes with musical aptitude [71] and is fundamental for inner ear development [134], the inferior colliculus, and in the determination of GABAergic neurons in the midbrain [135]. In addition, several targeted genes of *GATA* transcription factors resulted upregulated, indicating their probable main role in the alteration of gene expression [127]. The activity of *GATA* transcription factors is in turn indirectly determined by the activity of miR-222, which is upregulated after music performance [148]. SNCA, which is upregulated not only subsequently to music performance but also after listening to music [127,147], is involved in several functions, such as dopamine homeostasis (induces dopamine release), synaptic plasticity, as well as in neuropsychiatric and neurodegenerative diseases, such as Parkinson’s disease [130,131,132,133,153]. This finding is accordance with the increase of dopamine secretion in brain reward centers during listening to music, as seen using PET scanning [35].

Among the upregulated genes after music performance, there are target genes of *FOXP2*, towards which it acts as a transcriptional repressor [136]. Noteworthy, recent works showed that *FOXP2* is in turn targeted by different microRNAs upregulated after playing music (hsa-miR-93a-3p and hsa-miR-222-3p [148]; miR132 [150]). *FOXP2* (forkhead box protein P2) is a transcription factor which is involved in human language and speech development [137] and is associated with song learning and singing in songbirds [138,139,155]. Findings of upregulation of different microRNAs after music performance [126] support similar observations in songbirds, where these miRNAs are differentially regulated during song learning and listening [156]. The transcription of miR-222 is activated by *FOSL1* [157], which is expressed thanks to downregulation of hsa-miR-6803-3p after playing music [148]. It is also coexpressed with *FOS*, one of the top candidate genes associated with musical aptitude [111] and, in turn, upregulated after musical performance [127].

Songbirds show similarities with humans in this gene, in fact the corticostriatal *FOXP2* is regulated by miRNAs and *EGR1* [158], a gene which in turn is upregulated during song listening and singing in zebra finch [159]. This mechanism allows for adaptations of the brain structure for accurate song imitation [160].

Therefore, the clear involvement of *FOXP2* in music activities in humans, and in song production in songbirds, further supports a coevolution of music and language.

Moreover, among the upregulated microRNAs after music performance, there is hsa-miR-30d-5p [148], whose gene is located on 8q24.22 near the region previously associated with absolute pitch [123].

Both listening to and music performance induce a differential expression of microRNAs and genes involved in several functions, among which neuronal plasticity, learning and memory dominate. Both actions enhance the expression of genes involved in dopamine secretion and transport, coherent with the physical responses. Furthermore, the findings could support a convergence between music and language and even between sound perception and production in humans and songbirds.

## 7. Concluding Remarks and Perspectives

Music is an everyday presence in our lives and each of us like some form of it. The universality of music as a sociocultural component has prompted researchers to ask themselves if musicality is an adaptive characteristic with biological functions. Several different theories have been proposed, some regarding its evolutionary relationship with language, which remain a notable matter of debate among the scientific community.

In the attempt to define its origins, neuroimaging studies have shown that the biological foundations of music are inherent in brain functions, and playing any musical instrument requires neurocognitive functions, like learning and memory, a multicomponent integration of auditory and visual stimuli, and a high complex motor control [142,161]. Furthermore, several works have highlighted differences in neural circuits involved in rhythm perception between humans and non-human primates [68].

Moreover, several studies have been carried out to explicitly identify the genetic basis of musicality and dozens of genes have been so far associated with musical aptitude, as described in other relevant reviews by Oikkonen and colleagues [111], Järvelä [91] and most recently by Szyfter and Witt [162]. With the discovery that *FOXP2*, the gene previously regarded as the “language gene”, shows no prevalent signals of positive selection and is, therefore, not the paramount responsible for language evolution in humans, there is reason to believe that language actually arose as an extremely complex polygenic trait with strong influences from societal structures and cultural practices [163,164,165]. There could be a similar interpretation for the interplay of pleiotropic genes on strong cultural backgrounds in music ability [166,167,168]. However, in the last decades, most of the genomic studies have been carried out on Finnish population samples. The differences in genetic ancestry, ancient and recent admixture events that characterize all human populations, as well as prehistorical and historical sociocultural exchanges linked to instances of colonization, may have radically shaped and even changed the substrate on which the intersection of genetics and culture insists. Therefore, this review hopes to be a stimulus for novel genetic studies considering other populations, so as to highlight if there are clear worldwide differences in genes showing association with musical aptitude and to verify a possible universality of the genetic basis of musicality in humans. In fact, the inclusion of a diverse genetic background and comparative population studies could greatly benefit this highly interdisciplinary research field.

Evidence of functional analogy and sequence identity with several genes involved in vocal learning and singing in songbirds, mostly in the model species zebra finch [169], adds support to the idea of a convergent evolutionary event between bird vocalizations and human music as modulators of communication, even though further studies are needed to disentangle the interplay between genetics and social interactions in a cross-species perspective.

In a cultural setting, the intersection of music and speech can also generate new forms of art, such as song and poetry, that acquire specific structural characteristics from both language and music, and there is a tight cultural association between music, song and dance. The fields of poetry, human song and literary production, as fundamental cultural staples in human populations, could be more extensively analyzed from a genetic (possibly, polygenic) and interdisciplinary point of view. One interesting example is reported by a recent publication by Wassiliwizky and colleagues, in which it is demonstrated that, although poetry can elicit strong emotional responses in an analogous way as those observed when listening to music, the role of the nucleus accumbens and the whole neural circuitry appears different [170]. Moreover, the sharing of symbolic meaning and rhythm among speech, music and dance may be even better disentangled through novel transdisciplinary studies [171,172].

A final key aspect of musicality in humans that still has to be properly disentangled, however, pertains the presumed evolutionary advantages and related selective pressures that would justify the universal diffusion and conservation of such characteristic social behavior. In this framework, this comprehensive review suggests that a holistic approach integrating evolutionary genomics, archaeology and musicarcheology, ethnomusicology and musicology, and cultural anthropology may shed new light on the emergence, nature and evolution of social behaviors in human populations.

## Figures and Tables

**Table 1:** Main candidate genes for musical ability.

Gene	Chr Position	Association	Function	Refs.
***AVPR1A***	12q14.2	Dance performance, active listening to music, musical aptitude and creativity	Memory, learning, social behaviors	[93,96,100,101,102,103]
***SLC6A4***	17q11.2	Dance performance, short-term music memory	Implicated in emotions, neuropsychiatric disorders and depression	[96,101,105,106,107,108,109]
***UNC5C***	4q22.3	Musical aptitude	Axon extension, cell migration in developmental nervous system	[92,111]
***UGT8***	4q23	Musical aptitude	Complex lipids synthesis in myelinating oligodendrocytes, ceramides clearance in neurons	[98,114]
***PCDH7***	4p15.1	Musical aptitude	Cochlea development in chicken, post-natal and adult amygdala	[115,116,117]
***GALM***	2p22.1	Musical creativity	Involved in serotonin release and binding potential of serotonin transporter in thalamus	[95,123,125]
***PCDHA 1–9***	5q31.3	Musical aptitude	Synaptogenesis, serotoninergic projections’ maturation, learning, memory	[95,120,121]
***SNCA***	4q22.1	Upregulated after musical performance and listening to music	Dopamine homeostasis, synaptic plasticity, Parkinson’s disease, song learning in songbirds	[126,127,128,129,130,131,132]
***GATA2***	3q21.3	Musical aptitude	Inner ear development, inferior colliculus development, determination of GABAergic neurons, expressed in dopaminergic neurons	[99,133,134,135]
***FOXP2***	7q31.1	Targeted by upregulated miRNAs after listen to and music performance	Language development, song learning and singing in songbirds	[136,137,138,139]

## Data Availability

Data sharing not applicable: no new data were created or analyzed.
